# Pharmacokinetic Principles and Their Application to Central Nervous System Tumors

**DOI:** 10.3390/pharmaceutics12100948

**Published:** 2020-10-06

**Authors:** Joelle P. Straehla, Katherine E. Warren

**Affiliations:** 1 Dana-Farber/Boston Children’s Cancer and Blood Disorders Center, Boston, MA 02115, USA; joelle_straehla@dfci.harvard.edu; 2 Koch Institute for Integrative Cancer Research, Cambridge, MA 02142, USA

**Keywords:** blood:brain barrier, metabolism, delivery, CNS penetration, drug exposure

## Abstract

Despite increasing knowledge of the biologic drivers of central nervous system tumors, most targeted agents trialed to date have not shown activity against these tumors in clinical trials. To effectively treat central nervous system tumors, an active drug must achieve and maintain an effective exposure at the tumor site for a long enough period of time to exert its intended effect. However, this is difficult to assess and achieve due to the constraints of drug delivery to the central nervous system. To address this complex problem, an understanding of pharmacokinetic principles is necessary. Pharmacokinetics is classically described as the quantitative study of drug absorption, distribution, metabolism, and elimination. The innate chemical properties of a drug, its administration (dose, route and schedule), and host factors all influence these four key pharmacokinetic phases. The central nervous system adds a level of complexity to standard plasma pharmacokinetics as it is a coupled drug compartment. This review will discuss special considerations of pharmacokinetics in the context of therapeutic development for central nervous system tumors.

## 1. Introduction

Increasing knowledge of the biologic drivers of central nervous system (CNS) tumors has expanded over the last decade, increasing opportunities for disease-specific, molecularly targeted treatments. For example, genomic analyses of diffuse midline gliomas—high grade pediatric CNS tumors with a particularly poor prognosis—have led to the identification of molecular subgroups and putative drivers of disease [1,2,3,4]. Building upon this understanding, in vitro testing against large compound libraries has led to the identification of several promising therapeutics for this tumor type [5]. Similar pipelines of molecular subgrouping and target compound identification are even more mature for glioblastoma, the most common malignant brain tumor in adults. Despite this, the vast majority of molecularly targeted agents trialed for diffuse midline glioma and glioblastoma have yet to demonstrate efficacy in clinical trials [6,7,8]. Traditional cytotoxic therapies continue to play an important role in the treatment of many CNS tumors, yet their efficacy against certain tumor types, including malignant gliomas, remains limited. Ultimately, these agents may be used in combination with molecularly targeted agents. The primary challenge in therapeutic development for CNS tumors is effectively translating preclinical findings to the clinic. In addition to identifying the patient population most likely to benefit, effective translation of a preclinically identified “active” agent requires determination of the optimal formulation, optimal dose, route, and frequency of administration. The goal is to achieve effective exposure of a drug at the tumor site for a long enough period of time and administered as frequently as necessary to maintain the pharmacodynamic effects. For CNS tumors, in particular, a number of factors prevent this goal and contribute to the lack of efficacy.

## 2. Major Limitations to the Effective Treatment for Central Nervous System Tumors

### 2.1. Tumor Heterogeneity

Tumors of the CNS are a highly heterogeneous group, with more than 100 histologically distinct types of primary tumors that can arise throughout the brain, spinal cord, choroid plexus and meninges [9]. Classification of these complex tumors takes into account histologic features and anatomic location, and increasingly includes the presence or absence of certain molecular and genetic features [10]. Within an individual tumor, there is additional heterogeneity from cell to cell, as has been elucidated from single cell ribonucleic acid (RNA) sequencing of primary tumors [11]. The anatomic site of a tumor is critically important for treatment and prognosis, as surgical resection remains a mainstay of therapy for CNS tumors. The tumor microenvironment, influenced by underlying tumor biology as well regional differences in CNS architecture, is likewise highly variable. There also appears to be geographic variability of the blood:brain barrier within the CNS [12].

### 2.2. Inability to Assess Drug Concentrations over Time at the Tumor Site

Simplified models of pharmacokinetics that are reliant on serial blood sampling have long been used to determine dosing parameters of therapeutic agents. However, traditional pharmacokinetic models are not able to infer brain tissue concentration in a clinical setting. Many preclinical and some clinical studies have been undertaken to better understand and predict CNS pharmacokinetic properties of individual agents using direct or indirect measures of drug concentration in the CNS, but a comprehensive understanding of CNS pharmacokinetics in the setting of both normal and tumor tissue has thus far not been achieved. Single timepoint drug concentrations have been obtained at biopsy or surgical resection; while this may indicate whether a drug is able to enter the CNS, it does not characterize the exposure (concentration over time) at the tumor site.

### 2.3. Insufficient Methods to Evaluate Drug Effects on the Central Nervous System

Beyond the challenge of quantifying active drug concentration over time in the CNS, there remains the difficult task of determining drug effects in the CNS. In order to determine the pharmacodynamic (including on- and off-target) effects of an agent, there must be a known and measurable biologic endpoint. Pharmacodynamic effects are generally directly related to drug concentration, but other factors such as receptor density, drug affinity, ligand binding reversibility, residence time, or regulatory factors are also influential. Current methods to determine pharmacodynamic effects generally require tissue for biologic assays, though the emerging field of imaging biomarkers seeks to leverage non-invasive imaging techniques such as positron emission tomography (PET) imaging to enable clinical pharmacodynamic assessment [13,14].

### 2.4. Dynamic Nature of the Central Nervous System

While the CNS is often depicted as a static system for the purposes of modeling, it is actually a dynamic collection of neural and vascular networks that interact closely with brain structures to regulate blood flow and metabolic activity [15]. The system requires exquisite control of both flow and pressure through the vascular and ventricular systems. Significant variation in blood flow and metabolic activity occurs during exercise and throughout the sleep–wake cycle, both of which are influenced by aging [16,17]. Additionally, cerebrospinal fluid (CSF) is continuously produced and turned over; because there is no real barrier between the CSF and the extracellular space in the CNS, CSF and its turnover have an effect on the composition of the extracellular fluid and, therefore, the microenvironment of the brain [18]. These factors have been shown to have therapeutic implications; for example, circadian rhythms have recently been shown to moderate the effectiveness of the antiepileptic phenytoin through the downregulation of efflux transporters at night [19].

### 2.5. Pharmacokinetic Effects of Concomitant Medications

It has long been known that drug–drug interactions can lead to lack of efficacy or adverse effects. The most well-studied drug–drug interactions involve the cytochrome P450 (CYP) enzymes, a large family of hemoproteins that function in drug metabolism [20]. Co-administration a CYP family substrate with a drug of interest can lead to increased or decreased metabolism of that drug, and many commonly used medications are CYP substrates. There are also non-cytochrome P450 drug interactions, of which the role of drug efflux transporters is of particular interest in the CNS. Both P-glycoprotein (ABCB1) and breast cancer resistance protein (BCRP) are widely expressed in the blood:brain barrier, and modulation of the efflux transporter through the use of small molecule inhibitors has been employed in attempts to improve the CNS penetration of numerous anticancer agents [21,22,23]. Often, this results in higher systemic toxicity given the presence of these receptors in the liver and kidneys, and this approach has not led to greater efficacy of therapeutics against CNS tumors. Lastly, the use of medications such as dexamethasone and bevacizumab to control vasogenic edema in patients with CNS tumors is highly prevalent, but leads to reduced vascular permeability and has been linked to decreased efficacy of temozolomide [24,25,26,27].

### 2.6. The Blood:Brain Barrier

The blood:brain barrier (BBB) is intrinsically linked to central nervous system pharmacokinetics as it represents an important site of both absorption and elimination, as discussed in later sections. In addition, the heterogeneity of the BBB anatomically and within tumor-associated vasculature contributes greatly to the differential brain distribution of therapeutic agents. A description of the cellular and molecular components of the BBB is beyond the scope of this review, but has been reviewed in detail elsewhere in the literature [28,29,30,31,32].

## 3. Application of Pharmacokinetic Principles to the Central Nervous System

Pharmacokinetics is classically described as the quantitative study of drug absorption, distribution, metabolism, and elimination. The innate chemical properties of a drug, its administration (dose, route and schedule), and host factors all influence these four key pharmacokinetic phases. This review will discuss special considerations of pharmacokinetics in the context of therapeutic development for central nervous system tumors.

### 3.1. Absorption

**Figure 1:**
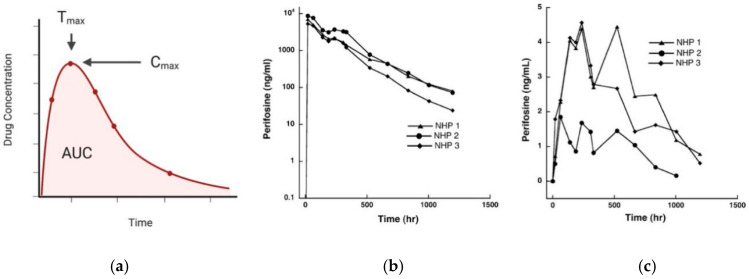
(**a**) Plotting the relationship of drug concentration and time defines the maximal concentration (C_max_) and time of maximal concentration (T_max_) for a given dose and route administration; (**b**) Experimental data showing drug concentration vs. time plots for perifosine in plasma and (**c**) Cerebrospinal fluid (CSF) after oral dosing to three non-human primates (NHP). Images (**b**–**c**) re-printed with permission from Cole et al. [33].

In the context of central nervous system pharmacokinetics, absorption is defined as the movement of a drug into the CNS. Similar to other regions of the body, two key determinants of CNS absorption are drug concentration in the bloodstream and the degree of protein binding. In traditional pharmacokinetic models, drug bloodstream concentration is plotted over time to determine parameters such as the maximal concentration (C_max_) and time of maximal concentration (T_max_) for a given dose and route of administration (Figure 1a). Plotting drug concentration in cerebrospinal fluid or brain tissue using a drug concentration vs. time plot highlights differential patterns of absorption into the CNS, as exemplified by experimental data shown in Figure 1b,c. While the principles and terminology are analogous to the classic plasma system, quantifying absorption is more complex in the CNS due to the dynamic relationships between drug concentration in plasma, cerebrospinal fluid, interstitial fluid and brain tissue. Achieving an adequate drug exposure at the site of action is essential to achieve a therapeutic effect, yet protein binding either in plasma or tissue can significantly hamper these effects as only free, unbound drugs can significantly cross the BBB to enter the CNS.

There are several additional factors that influence drug absorption into the CNS and to CNS tumors in particular. First, transport into the CNS occurs across two primary barriers: the blood:brain barrier and the blood:CSF barrier. For either barrier, this transport may be passive or active. Lipophilic molecules with low molecular weight can diffuse directly through cell membranes, however, the abundance of efflux pumps on both endothelial cells and choroid plexus epithelial cells severely limits transport in this manner [29,30,34]. Other molecules like glucose, amino acids, and some regulatory proteins pass the blood– and CSF–brain barriers via active transport, making use of symporters or transporters present in the endothelial cells of the blood–brain barrier or the epithelial cells of the CSF:brain barrier [28,32,35]. However, the majority of compounds with therapeutic potential for CNS tumors are polar, small molecules that cannot cross these barriers passively or actively in their native forms. To further complicate matters, expression of efflux pumps and transporters varies based on geographic location and also fluctuates dynamically in response to environmental cues, making it difficult to predict the relative absorption of a given molecule into the CNS.

### 3.2. Distribution

In the classical pharmacokinetic model, distribution refers to the rate of drug accumulation throughout all compartments, including central and peripheral compartments; in contrast, we will focus on drug movement within the central nervous system, including tumor accumulation. Once a drug has entered the CNS, its distribution depends on physiologic fluid movement, extracellular–intracellular exchange, and tumor-associated factors. Molecules that have passed across the blood:brain and/or blood:CSF barriers must then partition into the aqueous environment between cells. Thus, while lipophilicity is a primary determinant of blood–brain barrier permeability and hence *absorption* into the CNS, subsequent brain *distribution* is determined by hydrogen bonding potential; molecules with low hydrogen bonding potential are better able to distribute within extracellular fluid (ECF). The ECF-filled channels between cells are important for neuronal function and intercellular communication, and also represent a barrier to drug distribution. The composition of ECF is similar to CSF with the addition of negatively charged glycosaminoglycans and proteoglycans. These negatively charged polymers can limit the diffusion of molecules through both steric- and charge-dependent interactions [36]. In addition to diffusion, the recent discovery of glymphatic-mediated bulk flow of CSF into ECF and its role in solute clearance likely has implications for drug distribution as well as elimination, though much research remains to be done in this area [37]. Once in the extracellular space, molecules can also enter brain parenchymal cells through passive or active mechanisms, potentially leading to accumulation within a particular cell type. These first two factors of fluid movement and cellular exchange severely limit the distribution of most drugs, with diffusion rarely extending more than a few millimeters from the site of drug entry into the CNS.

Tumor-associated factors can lead to enhanced or impaired drug distribution in the CNS. For example, vasogenic edema leads to increased interstitial fluid pressure, thus limiting drug movement into tumors. Hydrocephalus, and resulting increased intracranial pressure, can limit drainage, potentially prolonging the residence time of a drug that has entered the parenchyma from the vascular system. Other factors such as ischemia and acidosis can limit drug distribution as they are intrinsically linked to lower blood flow to the tumor region. The impact of tumors on the local blood:brain barrier plays a significant role in drug distribution, though the common notion that the BBB is disrupted in all CNS tumors is overgeneralized. In fact, tumor-associated vasculature is highly heterogeneous within individual tumors and also among tumor subtypes. For example, the WNT subtype of medulloblastoma has been shown to have a particularly “leaky” BBB due to paracrine signals that block endothelial WNT signaling, whereas other subtypes of medulloblastoma have relatively intact BBB; this correlates with improved outcomes in the WNT subtype [38].

### 3.3. Metabolism

Metabolism is often defined as the process of enzymatically breaking down a drug into less active components to aid elimination from the body. However, there are some cases in which metabolism leads to more active compounds, either after the administration of a pro-drug or through the production of active metabolites. These principles of metabolism apply similarly to classical plasma pharmacokinetics as well as CNS-specific therapies. The three primary reactions involved in drug metabolism are oxidation–reduction, hydrolysis, and conjugation. As noted above, CYP family enzymes are the primary contributor to drug metabolism, and act to catalyze oxidation–reduction reactions. Although most CYP enzymes are predominantly expressed in the liver or lung, some are also expressed in the brain, primarily located in and around the cerebral blood vessels [39]. One of these, CYP2B6, is involved in the metabolism of the alkylating agents cyclophosphamide and ifosfamide to their active metabolites [20]. There are additional metabolic enzymes implicated in CNS drug metabolism, including several glutathione S-transferases and catechol O-methyltransferase, which all have higher expression in brain than liver [40].

### 3.4. Elimination

For CNS pharmacokinetic purposes, elimination simply refers to drug movement out of the CNS. This is analogous to classical pharmacokinetics in which elimination refers to drug movement out of the body, and can occur in several ways. First, a drug may enter into a cell or bind irreversibly to a cell surface receptor, effectively removing it from further action. Second, metabolic clearance occurs if the drug is degraded into inactive components within the CNS. Third, active drug can be physically removed from the CNS into blood or cerebrospinal fluid; this can occur through direct efflux into these fluids or via the glymphatic system. Through the glymphatic system, CSF distributes across brain parenchyma in perivascular spaces as extracellular fluid (ECF), functioning both to deliver CSF constituents to the brain parenchyma and also to clear parenchymal substrates, including drugs and metabolites. The current understanding of this process involves a bulk flow from CSF to ECF, then back to CSF, with eventual clearance into the bloodstream through subarachnoid granulations, meningeal lymphatics, and perineuronal spaces [36,37]. The baseline high rate of CSF turnover plays an important role in drug elimination, leading to a physiological sink in which CSF drug concentration is inherently lower than plasma concentration [41]. Hence, any alterations in CSF turnover can dynamically affect pharmacokinetics. For example, commonly used medications such as acetazolamide and omeprazole both decrease CSF production [42], and the presence of CNS tumors may lower turnover through obstruction of CSF flow through the ventricular system.

## 4. Implications for Central Nervous System Tumor Therapeutic Development

**Figure 2:**
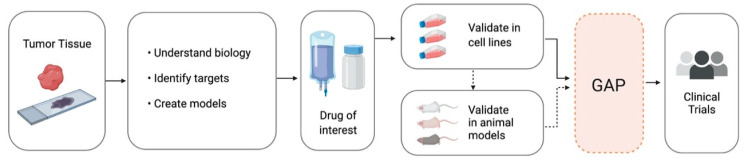
The current pipeline of drug development for central nervous system (CNS) tumors begins with deriving biologic information, therapeutic targets, and models from patient tissue. Then, drugs of interest are identified and validated in cell lines and (ideally, though not always) animal models before moving on to human clinical trials. There is a notable gap in knowledge regarding the ideal dose, route, and schedule for new drugs prior to starting clinical trials. Figure created with BioRender.com.

The current therapeutic development pipeline for CNS tumors follows a traditional “pan-cancer” approach, and often there is little emphasis on pharmacokinetics until the point of clinical trial development. The largest gap in knowledge and hence roadblock to effective clinical translation stems from the inability of current models to accurately predict the performance of a drug in clinical trials (Figure 2). This has led to frequent negative clinical trials, often without the ability to determine how or why an agent failed.

### 4.1. Clinical Attempts to Achieve Therapeutic Drug Concentrations at Tumor Site

**Figure 3:**
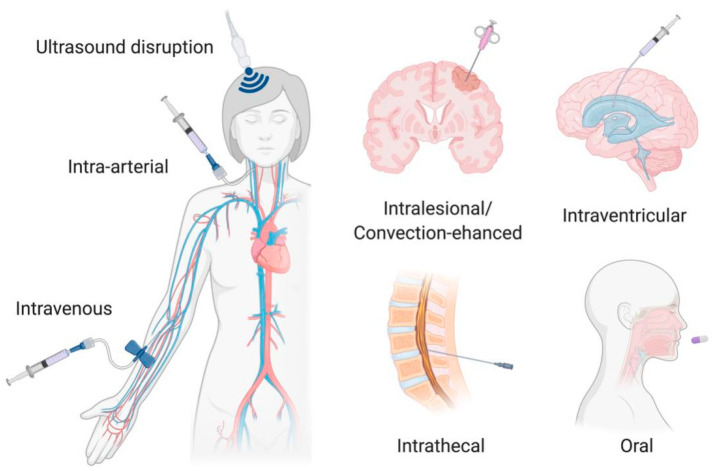
Administration routes and adjuvant technologies in current use for treatment of CNS tumors. Figure created with BioRender.com.

Very few therapeutically relevant drugs achieve adequate tissue concentration in the brain at doses that are standard for extracranial solid tumors. Therefore, numerous attempts have been made to improve drug delivery to CNS tumors. These include alternative administration routes and/or the use of adjuvant technologies (Figure 3). This review will focus on methods in current clinical use or late phase clinical trials, noting that many additional drug delivery strategies are in preclinical development or early phase clinical trials.

#### 4.1.1. High Dose Systemic Delivery

High dose systemic chemotherapy is often employed to obtain higher tissue concentrations in the brain and spinal cord. The principle underlying this approach is that if a constant fraction of a drug crosses the BBB, a higher dose will result in a higher tissue concentration. This has been shown to be true for some agents, though the relationship is often not linear and significant interpatient variability exists. For other agents, there appears to be a ceiling effect, with increased clearance rates tempering any clinical benefit. This has proved an effective strategy in certain situations; for example, the use of high dose chemotherapy with autologous stem cell rescue for young children with medulloblastoma has allowed some patients to avoid or delay craniospinal radiation [43]. The increased toxicity of these regimens is significant, however, and includes both therapy-related deaths and long-term toxicities.

#### 4.1.2. Regional Delivery

There are two prominent strategies that have been trialed to deliver therapeutics into a specific region of the brain preferentially: intra-arterial administration and blood:brain barrier disruption with focused ultrasound. Intra-arterial administration has the advantage of bypassing first pass metabolism through the venous system, such that a higher initial concentration of drug is supplied to the CNS. However, this pharmacokinetic advantage only applies to the first pass of the agent through CNS vessels; once the drug enters the systemic circulation, there is no longer any pharmacokinetic advantage. Given the rapidity of blood flow, the amount of drug able to pass from the CNS vasculature into the tumor is therefore limited. The most successful applications of intra-arterial chemotherapy have been in the treatment of peripheral malignancies such as retinoblastoma and hepatic tumors, but it has also been utilized for high grade CNS tumors and remains an active area of clinical research. Intra-arterial administration can also be combined with agents to enhance BBB opening such as mannitol, which transiently opens the tight junctions of the BBB. For example, Zylber-Katz et al. collected CSF and serum data from patients with CNS lymphoma following administration of methotrexate via intra-arterial (with or without mannitol) or intravenous delivery (without mannitol) and reported higher C_max_ in the CSF and higher CSF/serum ratios for patients receiving mannitol and intra-arterial administration [44]. Intra-arterial administration with BBB disruption also increases the potential for neurotoxicity, limiting the number of agents amenable to this administration method [45].

Focused ultrasound has been increasingly combined with systemic drug administration to improve regional drug delivery. By directing low intensity ultrasound to the region of the tumor in combination with the administration of microbubbles, transient and local BBB disruption is achieved via the cavitation effect [46]. While numerous technologies are under investigation for clinical use, two that have progressed furthest in clinical trials are magnetic resonance guided focused ultrasound (MRgFUS) and implantable low-intensity pulsed ultrasound devices. Both methods have recently shown safety and feasibility of repeated BBB disruption in adult patients with high grade gliomas when combined with chemotherapy [47,48]. Though trials to date have not focused on quantitative pharmacokinetics, Mainprize et al. recently reported data from two patients receiving MRgFUS with intravenous liposomal doxorubicin and oral temozolomide, respectively, prior to scheduled tumor resection. In both patients, there was a trend toward higher drug concentration in sonicated compared to non-sonicated peritumoral tissue analyzed after resection [49]. As the field continues to expand, research is focusing on optimizing dosing parameters (ultrasound pressure, therapy schedule and duration, timing of drug administration in relation to microbubbles and ultrasound), controlling the field of BBB disruption, and determining which therapeutic agents are best suited to pair with focused ultrasound. Tumor location remains an additional limiting factor; depending on the technology, deep-seated tumors such as those in the brainstem may be too far away from the ultrasound source to achieve the desired effects [50,51].

#### 4.1.3. Intrathecal and Intraventricular Delivery

Another strategy to increase tumor drug concentration is direct injection of therapeutic agents into the cerebrospinal fluid through a lumbar puncture or the use of an intraventricular reservoir. Unfortunately, the parenchymal penetration of drugs administered in this manner is only a few millimeters, severely limiting its application in most parenchymal-based tumors [52]. However, intrathecal and intraventricular chemotherapy plays an important role in tumors with leptomeningeal seeding, as well as in hematopoietic malignancies with CNS involvement [53]. Some challenges with this modality include the limited number of drugs with acceptable side effect profiles and heterogeneous CSF drug concentrations at different sites due to CSF circulation and mixing patterns. Additionally, it is difficult to maintain effective concentrations for prolonged periods of time given CSF clearance rates. In the clinical setting, Blaney et al. reported results from a phase I pharmacokinetic study highlighting the importance of optimal dosing studies to determine the appropriate dose and schedule of intraventricular topotecan within a specific clinical context [54]; while this did not translate to improved patient survival in their cohort of pediatric patients with neoplastic meningitis, the work nonetheless brings important insight to the field.

#### 4.1.4. Local Delivery

The ability to directly deliver a drug to CNS tumors using convection enhanced delivery (CED) has been studied in preclinical models and shown to be safe and feasible clinically, with the first human trial data published over 20 years ago [55]. The underlying principle for CED is that a low, continuous positive pressure infusion will move macromolecules via convection, enhancing the volume and homogeneity of tissue drug distribution in the brain and effectively bypassing the BBB [56]. Early CED trials using “off-the-shelf” catheters placed stereotactically into CNS tumors suffered from logistical and technical limitations leading to heterogeneous tissue distribution, but the development of specialized tools and surgical planning software has renewed interest in this administration strategy [57]. Traditionally, co-infusion of the contrast agent gadolinium has been used to infer drug distribution, though this may not be true for all therapeutic agents. Recently, Souweidane et al. reported safety and feasibility results from a phase I trial in children with diffuse intrinsic pontine glioma, delivering a theranostic radiolabeled antibody that allowed simultaneous tracking of volume of distribution and dosimetry [58]. There remain several obstacles to overcome with CED, including finding a standardized measure of volume of distribution, determining which therapeutic agents are most amenable to CED, optimizing treatment regimens to achieve therapeutic concentrations for adequate lengths of time to improve patient outcomes, and defining tumor target volume, particularly for invasive tumors.

### 4.2. Defining Effective Exposure

Regardless of the route of drug administration used, the key determinant of clinical response to a therapeutic agent is *effective exposure* at the tumor site, though this remains difficult both to define and to measure. For traditional chemotherapeutic agents, traditional in vitro dose–response curves are often used to define a minimum effective dose for a given tumor type. For example, the half-maximal inhibitory concentration (IC50) determined from cell growth assays is often used as a benchmark when looking at tissue drug concentration. However, there are limitations to this strategy, as in vitro continuous exposure does not recapitulate the complex tumor microenvironment in which metabolism and elimination lead to heterogeneous and dynamically changing drug levels. The IC50 was predicated on the premise that for cytotoxic chemotherapeutic agents, there is a dose–response relationship. Determining an effective dose of molecularly targeted agents poses a more difficult task, as their mechanisms of action may not be dose-related and may be difficult to capture in traditional in vitro assays. In these cases, attention to pharmacodynamic parameters and their correlation to drug concentration over time becomes particularly important. This concept is further discussed below in Section 4.3.

In order to determine whether an effective exposure has been attained, an accurate measure of drug concentration over time is critical. Several strategies have been implemented to measure CNS tumor drug concentration, each with its benefits and drawbacks (Table 1). First, CSF sampling has been used as a surrogate measure of tumor drug concentration. This has the advantage of being more readily attained than tissue, with the potential for sampling at numerous timepoints to generate a curve for modeling; however, the ability to directly extrapolate to tumor tissue concentration remains limited. As discussed above, active drug in the CSF still has numerous barriers to cross in order to distribute into the interstitial space, and in many cases is rapidly cleared back into the CSF. Second, direct tumor sampling via biopsy or autopsy sample may provide an accurate measure of drug concentration, but requires an invasive procedure, may be contaminated with blood, and, in almost all cases, only a single timepoint is possible. The surgical or autopsy sample must be timed in relation to an administered dose, and high interpatient variability limits the extrapolation of these samples to a broader patient population [59]. Third and least invasive, positron emission tomography (PET) imaging allows for quantification of drug concentration in the human body with some spatial resolution. However, in order to use this method, a radiolabeled version of a drug must be developed, which is time- and labor-intensive, and will not be possible for all molecules of interest [13,14]. Lastly, the gold standard but least accessible method is microdialysis. This technique allows for the determination of local drug concentration in a tissue or fluid using a probe inserted into the tissue of interest. By sampling the dialysate, the concentration of drug in the interstitial space can be inferred, though modeling must take into account tissue diffusion properties and probe calibrations, preferably in the tissue being sampled. Microdialysis has been an underutilized but critical component of preclinical research in CNS pharmacokinetics and has played a role in select clinical trials, but because it is invasive, expensive, variable, and requires highly specialized equipment and skill, it remains difficult to implement in clinical settings [60].

### 4.3. The Path Forward

In order to make progress in therapeutic development for CNS tumors, we must follow the principle of achieving effective exposure of a drug at the tumor site for a long enough period of time, and administered as frequently as necessary, to maintain the pharmacodynamic effects. This will require an integration of pharmacokinetics and pharmacodynamics, drawing iteratively upon both preclinical studies and clinical trial data to develop predictive models [61]. Ultimately, an improved understanding of these principles may allow for the development of novel agents with optimized pharmacokinetic properties for the CNS.

#### 4.3.1. Expand Preclinical Testing to Develop Integrated Pharmacokinetic Models

Preclinical in vivo testing is often aimed at validating targets previously identified from tissue- or cell culture-based investigations. As such, many novel therapeutic agents are tested in a single animal model, at a limited dose range via a single administration route, using an arbitrary schedule. These studies often have endpoints such as median survival time with secondary biologic analyses of tissue to determine the effect of the investigational agent, generally at a single point in time. While these studies are useful in validating therapeutic targets, they do not provide sufficient information for clinical trial study design. Similarly, traditional preclinical studies undertaken specifically for pharmacokinetic analyses are useful in determining the systemic pharmacokinetic parameters such as plasma half-life and clearance routes of a drug at various dosing regimens and may also inform toxicity, yet are not generally designed to provide necessary pharmacodynamic information.

Ideally, additional preclinical testing would take place after target validation and systemic pharmacokinetic principles have been investigated with the specific aim of determining a dose, route, and administration schedule that achieves effective exposure at the tumor site. To do this, drug–target interactions must be rigorously explored both in vitro and in vivo. Drug–target interactions are defined by both the strength of interaction (the drug–target dissociation constant K*_d_*) and the drug–target residence time (the inverse of the first-order dissociation rate constant, or 1/*k*_off_) [62]. This concept of drug–target residency time is particularly important for targeted small molecule therapeutics, and must be considered together with target occupancy, as both contribute to the duration of clinical effect and will inform dose and administration schedule. While these studies are both time- and cost-intensive, the development of an integrated pharmacokinetic and pharmacodynamic model will require both experimental inputs and computational expertise.

#### 4.3.2. Include Pharmacokinetic and Pharmacodynamic Endpoints in Clinical Trials

In order to inform and improve preclinical models, it is imperative that clinical trials provide data that can directly feedback to preclinical investigators. As such, clinical trials must be undertaken carefully, with goal of determining not just whether an agent is effective in a given patient population, but also validating *why* it is working—or more often, why it is *not* working.

This necessitates the incorporation of simultaneous pharmacokinetic and pharmacodynamic measures in clinical trials, a difficult task given the limitations expounded in this review. This may necessitate flexibility in enrollment criteria to allow for drug administration prior to tumor resection, such that tissue collected at the time of a medically necessary surgery can be meaningfully analyzed for drug content and pharmacodynamic effect. While plasma and CSF drug levels alone are not sufficient to predict tumor concentration, judicious gathering of these samples may inform preclinical models, especially if obtained simultaneously at the time of any tumor tissue collection. Notably, to be clinically applicable and informative, this must occur in the setting of a *therapeutic* dose, as opposed to the traditional notion of a phase 0 trial in which a subtherapeutic dose of a novel agent is given prior to a planned surgery.

#### 4.3.3. Development of Agents that Distribute across the CNS

Ultimately, there is hope that an improved understanding of CNS pharmacokinetics will inform the pipeline of drug development as well as the repurposing of existing drugs using new or revised dosing strategies that have been thoroughly vetted in preclinical models. As new agents with improved CNS distribution and residence time are developed, potential off target effects will need to be considered, especially in certain patient populations such as the very young or the elderly. In the preclinical setting, this may involve investigating the effects of targeted agents in the setting of brain development and over long periods of time.

## 5. Conclusions

An understanding of CNS pharmacokinetics is essential to improve outcomes for patients with CNS tumors. There are many limitations to effective drug delivery within the CNS, and our understanding of absorption, distribution, metabolism and elimination remains incomplete. Tumor-specific factors, driven by the heterogeneity and diversity of CNS tumors both biologically and anatomically, add complexity. To spur progress in this area, an integrated approach to studying pharmacokinetics in concert with pharmacodynamics is needed, including bidirectional data from computational simulations, preclinical models, and clinical trials.

## Figures and Tables

**Table 1:** Methods to assess drug concentration at tumor site, highlighting advantages and disadvantages.

Method	Advantages	Disadvantages
CSF sampling	Relatively noninvasive; Sampling at multiple timepoints possible	CSF concentration is a proxy for tumor interstitial fluid
Direct tumor sampling (via biopsy/autopsy)	Provides direct sampling of tumor interstitial fluid	Highly invasive; Logistically difficult to time with drug dose; Generally single timepoint
Positron emission tomography (PET) imaging	Noninvasive; Sampling at multiple timepoints possible	Requires radiolabeled version of drug; Extensive validation necessary prior to clinical implementation
Microdialysis	Provides direct sampling of tumor interstitial fluid; Sampling at multiple timepoints possible	Highly invasive; Expensive, requires specialized equipment and skill
